# Does insomnia increase the risk of suicide in patients with major depressive disorders? national inpatient sample analysis

**DOI:** 10.1192/j.eurpsy.2021.453

**Published:** 2021-08-13

**Authors:** A. Reddy, Z. Mansuri, R. Vadukapuram, M. Thootkur, C. Trivedi

**Affiliations:** 1 Psychiatry, Virginia Tech Carilion School of Medicine, Roanoke, United States of America; 2 Department Of Psychiatry, Boston Children’s Hospital/Harvard Medical School, Boston, United States of America; 3 Psychiatry, Icahn School of Medicine at Mount Sinai, New York, United States of America; 4 Research, St Davids Healthcare, Austin, United States of America

**Keywords:** Insomnia, Depression, Suicide, mood disorders

## Abstract

**Introduction:**

Insomnia is strongly associated with Major depressive disorders (MDD). There is strong evidence that it is one of the risk factor for suicide. Studies have shown the relationship of suicidal behavior in MDD patients with insomnia. However, it has not been evaluated in a large inpatient sample.

**Objectives:**

To evaluate suicidality in MDD patients with insomnia compared to those without insomnia.

**Methods:**

From the National Inpatient Sample (NIS 2006-2015) database using ICD-9 code, we obtained patients with the primary diagnosis of MDD and comorbid diagnosis of insomnia disorders (MDD+S). We compared it with MDD patients without insomnia disorders (MDD-S) by performing a 1:2 match for primary diagnosis code in the unweighted dataset. Suicidal ideation/attempt data were compared between the groups by multivariate logistic regression analysis.

**Results:**

After the diagnostic code matching, 139061 patients were included in the MDD+S group and 276496 patients in the MDD- S group. MDD+S patients were older (47 years vs 45 years, p < 0.001) compared to the MDD-S group. Prevalence of Suicidal ideation/attempt was 56.0% in the MDD+S group and 42.0% in the MDD-S group (p < 0.001). After adjusting for age, sex, and race, MDD+S was associated with 1.8 times higher odds of suicidal behavior compared to the MDD-S group. (Odds ratio: 1.79, 95% confidence interval 1.68-1.91, p < 0.001).

**Conclusions:**

Insomnia in MDD patients is significantly associated with the risk of suicide. It is important to be watchful for insomnia in MDD patients.

**Disclosure:**

No significant relationships.

